# Rabies Vaccine Characterization by Nanoparticle Tracking Analysis

**DOI:** 10.1038/s41598-020-64572-6

**Published:** 2020-05-18

**Authors:** Navarro Sanchez, D. Soulet, E. Bonnet, F. Guinchard, S. Marco, E. Vetter, N. Nougarede

**Affiliations:** Analytical Research & Development, Sanofi Pasteur, Campus Mérieux, 1541 Avenue Marcel Merieux, 69280 Marcy l’Etoile, France

**Keywords:** Infectious diseases, Vaccines

## Abstract

There are concerns that effectiveness and consistency of biopharmaceutical formulations, including vaccines, may be compromised by differences in size, concentration and shape of particles in suspension. Thus, a simple method that can help monitor and characterize these features is needed. Here, nanoparticle tracking analysis (NTA) was used to characterize particle concentration and size distribution of a highly-purified rabies vaccine (RABV), produced in Vero cells without raw materials of animal origin (RMAO). The NTA technique was qualified for characterization of RABV particles by assessing the stability profile of vaccine particles over 5–55 °C. Antigenicity of the viral particle was also monitored with the enzyme-linked immunosorbent assay (ELISA) and NTA. RABV particle size diameters were 100–250 nm (mean:150 nm), similar to sizes obtained when labelled with rabies anti-G D1–25 monoclonal antibody, suggesting mainly antigenic virus-like particles, also confirmed by transmission electron microscopy. Thermal stress at 55 °C decreased the concentration of anti-G D1–25-labelled particles from 144 hours, coherent with conformational changes leading to loss of G protein antigenicity without impacting aggregation. Results from RABV antigenicity assessment during the 24 months monitoring of stability showed good correlation between NTA and ELISA. NTA is a suitable approach for the characterization of biopharmaceutical suspensions.

## Introduction

Rabies is a fatal zoonotic encephalitis caused by lyssaviruses from the Rhabdoviridae family. Rabies virus infects a wide number of domestic and wild animal species worldwide and is transmitted to humans through the saliva of infected animals following bites or scratches^1^. The disease claims the lives of an estimated 60,000 people annually and remains an important worldwide health problem^2^. Since the first anti-rabies vaccination conducted by Louis Pasteur and Emile Rouxin 1885^3–5^, a number of different vaccines for use in humans have been developed, including those prepared in human diploid cells, Vero cells, and purified chick embryo cells^6^. There are currently 2 licensed vaccines manufactured by Sanofi Pasteur: Human Diploid Cell Rabies (HDCV, Imovax rabies) and Purified Vero Cell Rabies Vaccine (PVRV, Verorab). Sanofi Pasteur has improved PVRV to develop a next generation, serum-free, highly-purified Vero cell vaccine (PVRV-NG2, also called VRVg 2.0 in this publication).

It is vitally important to characterize vaccines during the production process in order to develop analytical protocols to ensure that potency and lot-to-lot consistency of the final product is monitored. The characterization of particulate matter in vaccine formulations is essential as aggregation may compromise safety and therapeutic efficacy^7–12^. Defined criteria for visible and sub-visible particles are included in pharmacopeias^13–17^.

There are many analytical techniques for assessing and monitoring particles in biopharmaceuticals. Nanoparticle tracking analysis (NTA)^18^ is a relatively new technique for the analysis of virion size, particle concentration, and aggregation state. NTA allows for the assessment of particles in the size range 10 nm and 1 µm, with real time visualization of individual particles enabling particulate suspensions to be characterized in greater detail under various conditions^19,20^. The technique has shown applicability in characterizing the size, concentration, and stability of a number of viruses including adenoviruses, influenza A, vesicular stomatitis virus, rabies (VRVg), hepatitis E virus-like particles preparations and therapeutic antibodies^21–24^.

Currently, the NIH test based on mice immunization followed by intracerebral viral challenge is used to assess the potency of rabies vaccines. As part of an initiative to replace animal-based tests by alternative methods, enzyme-linked immunosorbent assay (ELISA) has been successfully tested and suggested as a replacement for the NIH test^25^, but there is room for other non-animal-based techniques to support this replacement.

In this study, NTA was used to characterize Verorab and PVRV-NG2 (VRVg2.0, RABV) by particle quantification and size estimation. The stability of the particle size distribution over 5–55 °C was assessed by measuring the particle size distribution, aggregation, and antigenicity of the vaccine antigen. Particle antigenicity monitored with ELISA was compared with that measured by NTA to examine consistency of results across methods. Finally, the NTA method was qualified to indicate if it was suitable for the characterization of viral particles in vaccines in terms of specificity, linearity, accuracy and precision, with no matrix interference.

## Results

### RABV viral particle size and counting measured by NTA

**Figure 1:**
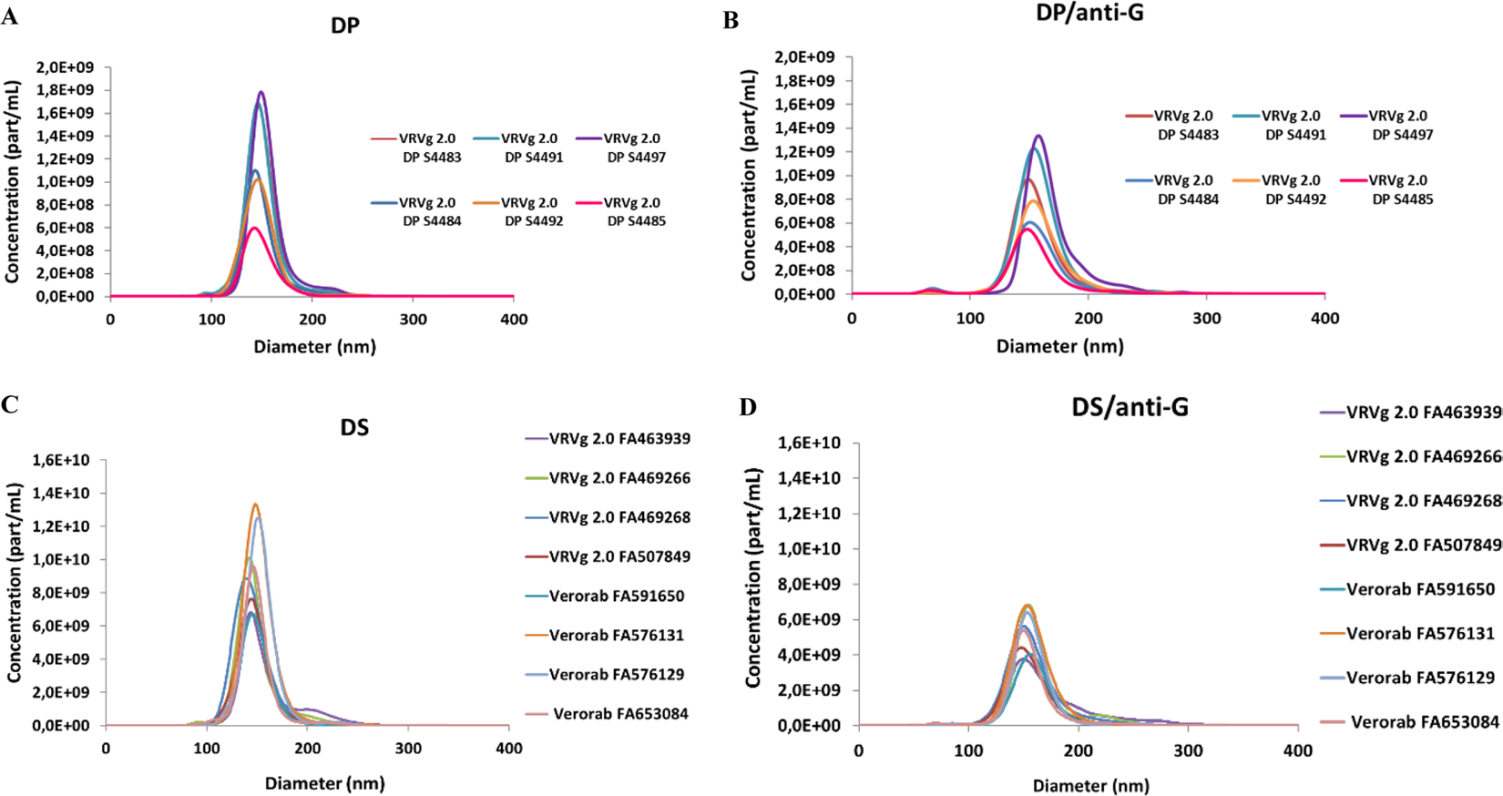
PVRV-NG2 (VRVg 2.0) batch particle size distribution by NTA. (**A**) DP without anti-G D1–25 labelling (**B**) DP with anti-G D1–25 labelling (**C**) DS without anti-G D1–25 labelling (**D**) DS with anti-G D1–25 labelling.

The particle size distribution of drug substance (DS) during the manufacturing process and final drug product (DP) batches were similar, with mono-modal profiles (Fig. 1A,C). Particle size diameters ranged from approximately 100 nm to 250 nm (the polydispersity reflecting different viral particle forms), with a mean population peak around 150 nm in agreement with the expected size of rabies virus family (approximately 60 nm by 180 nm)^26^, expressed as spherical equivalent diameter. The concentration of particles in the DP batches ranged from 1.88 ×10^10^ to 5.72 ×10^10^ particles/mL and those in the DS batches ranged from 1.65 ×10^11^ to 4.55 ×10^11^. The disparity in particle concentration within individual DP and DS batches is due to particles of different sizes within the sample. The discrepancy in particle concentration between DP and DS batches is due to differences in the formulation process which is based on ELISA. In addition, the DP has been formulated at different doses based on clinical studies (low, medium, high), the NTA technology (as well as ELISA) allows verification of the correct dose and Fig. 1 shows that the NTA can discriminate the three different doses with unlabelled and labelled particles. Similar results were obtained (in terms of particle size distribution and concentration) when RABV particles were labelled with rabies anti-G D1–25 monoclonal antibody (Fig. 1B,D) showing the particles were all specifically RABV. The successful labelling of the G protein epitopes suggests they were intact and mainly antigenic (confirmed by electron microscopy), demonstrating that NTA is able to quantify antigenic RABV particles, including at different states of manufacturing and different formulations. Analysis of three of the DP batches which were formulated at the same G protein content target by ELISA, demonstrated lot-to-lot consistency of both particle concentration and particle size distribution (Figure S1).

### RABV particle visualization by transmission electron microscope (TEM)

**Figure 2:**
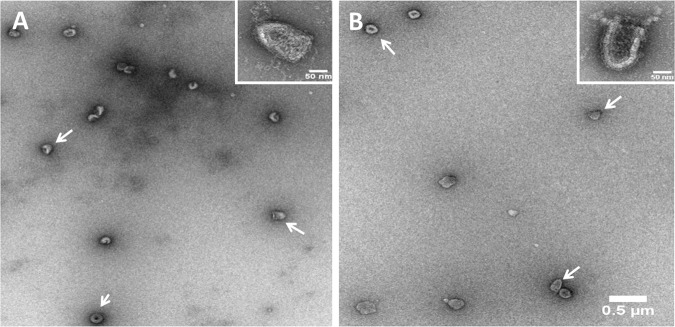
TEM micrographs of PVRV-NG2 (VRVg 2.0) particles. (**A**) Negative stained EM-field of RABV DP lot S4491. Particles appear to have a homogeneous bullet-shape uniformly distributed and not aggregated. (**B**) Negative stained EM-field of inactivated rabies virus after incubation with anti-G D1–25 monoclonal antibodies (arrows). Particles depict the canonical bullet-shape of RABV (insert panels show extracts from other micrographs of the samples), which is an International Committee on Taxonomy of Viruses (ICTV) taxonomic characteristic of the *Lyssavirus* genus to which RABV belongs.

The RABV particles were also visualized by TEM (Fig. 2). The particles were not aggregated but uniformly distributed irrespective of whether they were not labelled (Fig. 2A) or labelled with anti-G D1–25 monoclonal antibody (Fig. 2B).^27^ They appear as a homogeneous population of bullet-shaped particle corresponding to the expected taxonomic characteristic of *Lyssavirus* genus. These results confirm that the structure of the viral particle is not altered by the interaction with the monoclonal antibody, thus supporting the specific labelling of antigenic rabies virus particles required to qualify the NTA results.

### RABV drug product particle stability

**Figure 3:**
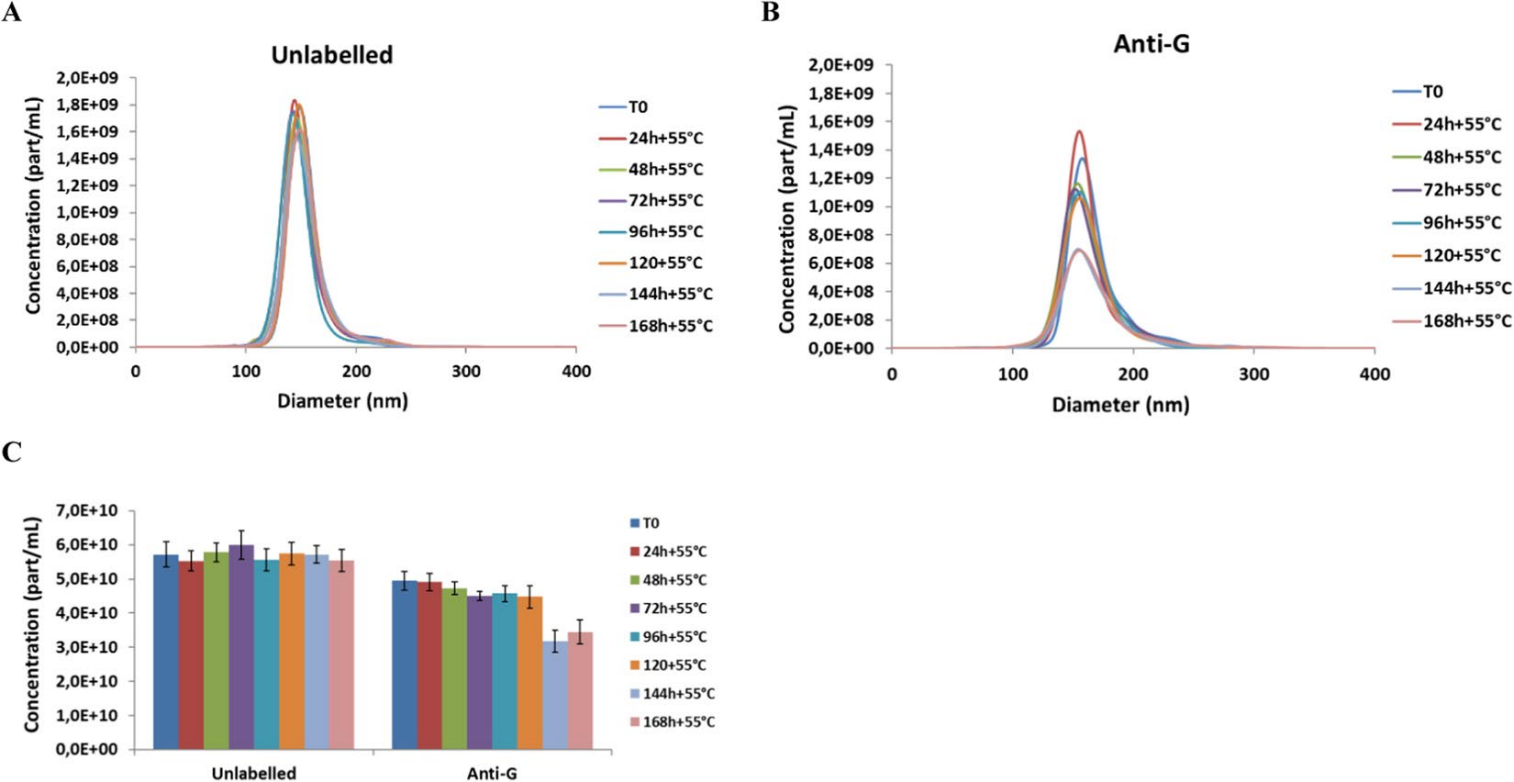
PVRV-NG2 (VRVg2.0) DP particle size distribution by NTA with thermal stress at 55 °C. DP lot S4497 was incubated at 55 °C for up to 7 days. Particle concentration and size distribution without labelling (**A**) and with labelling (**B**) with anti-G D1–25 monoclonal antibody. (**C**) Histogram representation showing the total concentration. Error bars represent standard deviation.

**Figure 4:**
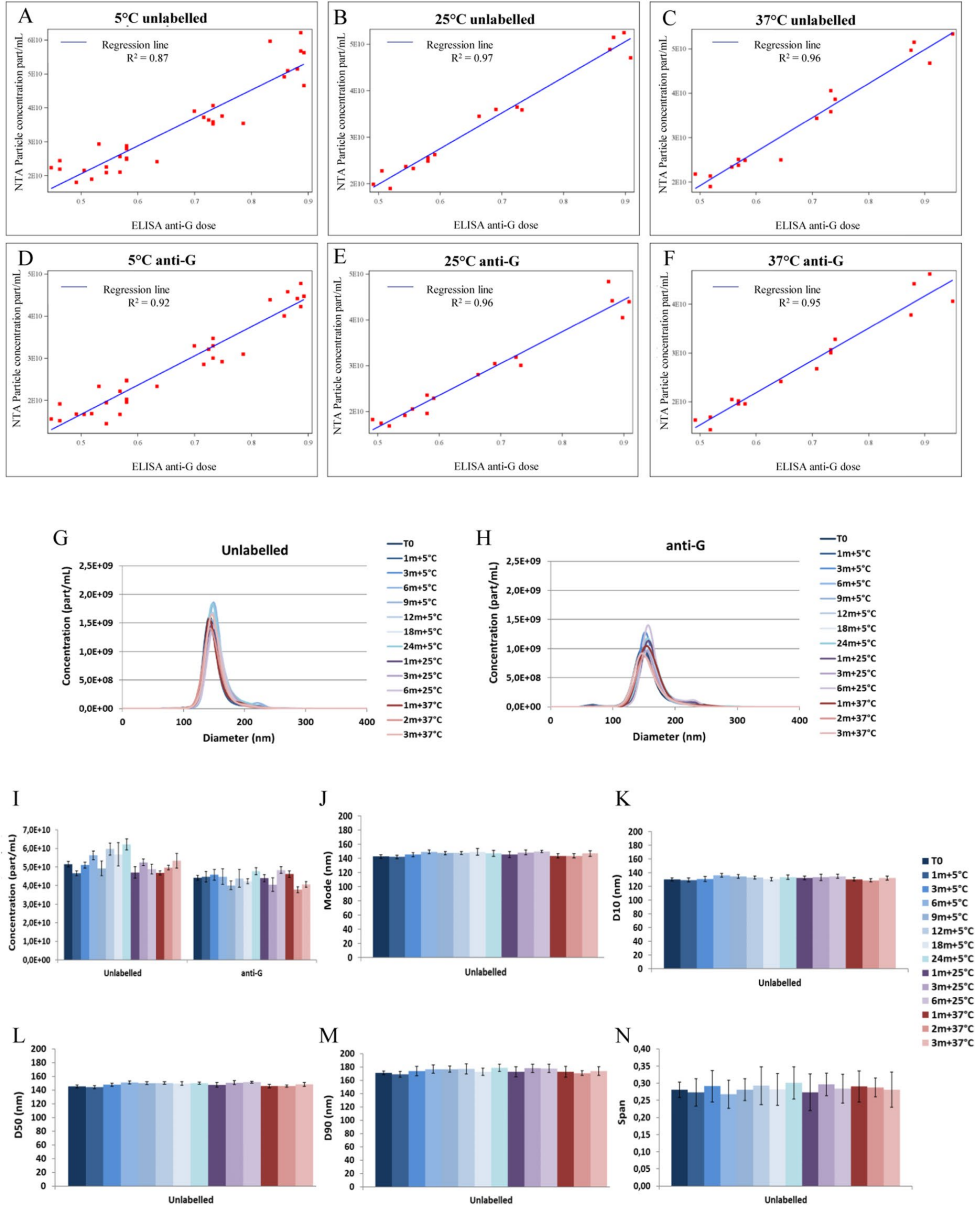
Labelled anti-G D1–25 monoclonal antibody labelled particle concentration vs antigenicity determined by ELISA: (**A**) 5 °C unlabelled (**B**) 25 °C unlabelled (**C**) 37 °C unlabelled (**D**) 5 °C anti-G (**E**) 25 °C anti-G (**F**) 37 °C anti-G. Each dot depicts a separate sample. Anti-G and size distribution determined by NTA: (**G**) 5–37 °C unlabelled (**H**) 5–37 °C anti-G. Each line depicts a separate sample measured at a specific month. Histogram representation: (**I**) Concentration (**J**) Mode (**K**) D10 (**L**) D50 (**M**) D90 (**N**) Span. Each bar depicts a separate sample measured at a specific month. Error bars represent standard deviation. All measured by PVRV-NG2 (VRVg 2.0) DP lot S4483 at 5–37 °C over 24 months.

Although the concentration of unlabelled particles remained relatively unchanged with thermal stress over one week (Fig. 3A,C), there was a decrease in the concentration of anti-G D1–25-labelled particles from 144 hours at 55 °C (Fig. 3B,C). Thermal stress had more of an effect on anti-G D1–25-labelled DP particles as the anti-G D1–25 monoclonal antibody recognizes the conformational glycoprotein epitope,. Thus, thermal stress induces significant conformational changes in the RABV particles, specifically changes that lead to a loss of G protein antigenicity over time without impacting the aggregation state. The decrease in RABV (DP) antigenicity with thermal stress was confirmed by ELISA which showed slightly more sensitivity than NTA anti-G D1–25-labelled samples (Figure S2). The particle concentration and size distribution (Fig. 4) determined by NTA were stable over 24 months at 5–37 °C. Unlabelled particles have no specific viral epitope targeted for antigenicity, therefore are used to verify the viral particle integrity/intactness under temperature stress.

### Correlation between NTA and ELISA

The monitoring of the stability of the RABV (DP) over 24 months at 5 °C, 6 months at 25 °C and 3 months at 37 °C confirmed the ability of the NTA to count and size antigenic RABV particles. There was a good correlation between antigenic RABV (DP) anti-G D1–25-labelled particle concentrations determined by NTA and ELISA (Fig. 4A–F). In addition to counting and sizing particles, as the ELISA, the NTA detected the rate of change of viral particle properties over time as a consequence of the exposure to temperatures higher than those recommended for storage, most likely accounting for the high correlation between methods (Fig. 4G,H). Furthermore, the constant size distribution and concentration of RABV over time as measured by mode, D10, D50, D90 and Span (Fig. 4I–N) showed that the viral particle was very stable.

### Particle concentration and size distribution qualification by NTA

**Figure 5:**
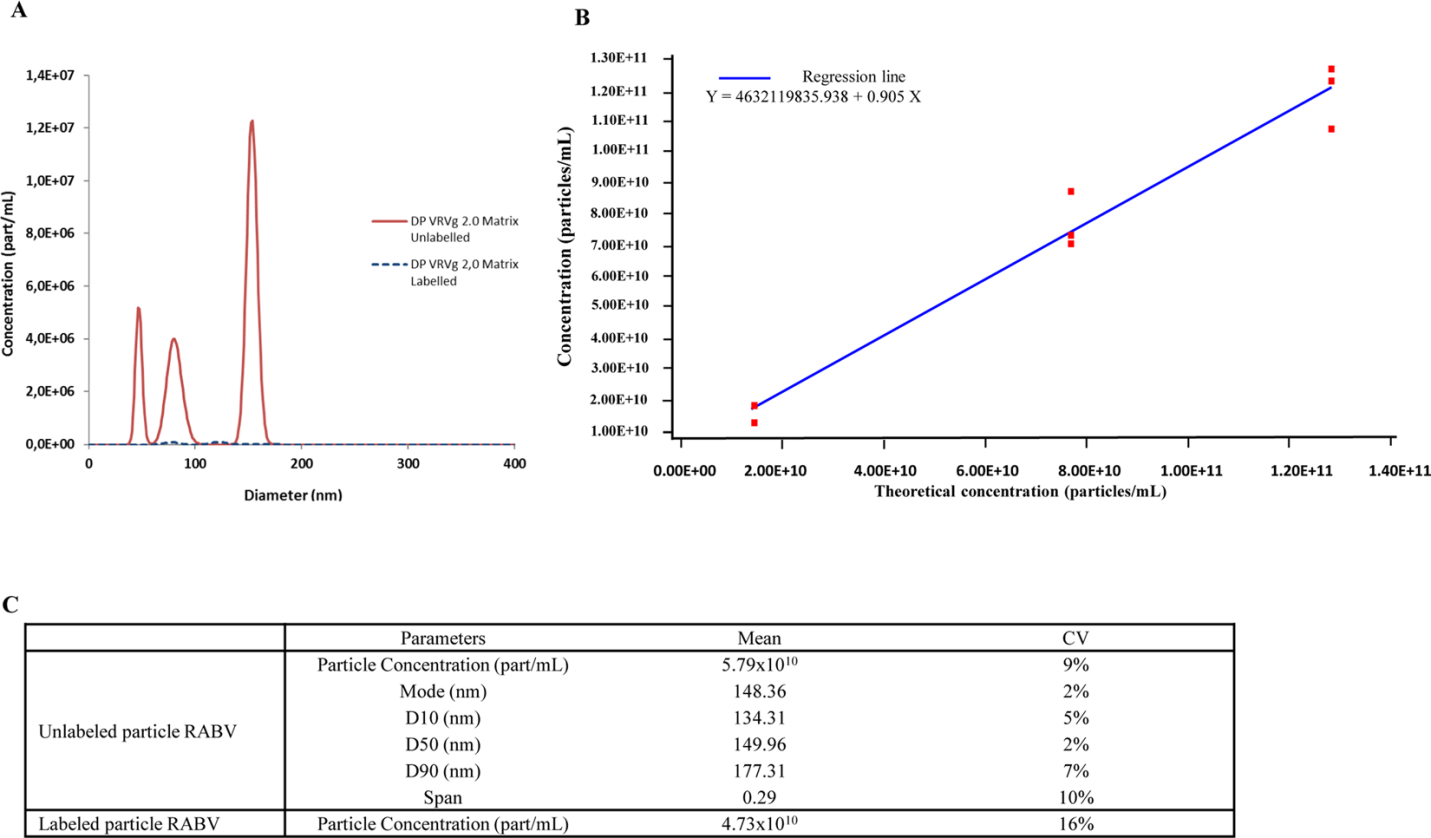
NTA technique qualification (**A**) Specificity: matrix only particle distribution measured by NTA (**B**) Linearity: anti-G D1–25 monoclonal antibody labelled particle concentration (**C**) Precision: mean of six NTA repetitions. All measured by PVRV-NG2 (VRVg 2.0) DP lot S4491.

To evaluate the performance of NTA to determine particle concentration and size distribution, the method was first qualified. The specificity was assessed using matrix alone (no antigen) and showed that the technique was specific since no RABV particles were detected with or without anti-G D1–25 labelling. The particle size distribution of the matrix-only samples had different peaks at different sizes (Fig. 5A), which were not in agreement with the expected size of rabies virus family, but inherent to those present in the diluent and considered negligible. The method is thus considered specific due to the absence of response of the matrices alone. The method was linear for the RABV labelled with anti-G D1–25 over the particle count range 1.62×10^10^–1.19×10^11^ with a corresponding coefficient of determination (R^2^) of 0.97 (Fig. 5B). Accuracy of measurement was demonstrated for the concentration in particles by an average recovery between (94% and 96%) for the unlabelled particles and between 93% and 115% for the anti-G D1–25 monoclonal antibody labelled particles. Thus, the method can be considered accurate. Precision was demonstrated as the coefficient of variation showed minimal variation between the six repeats of NTA runs for RABV labelled with and without anti-G D1–25 monoclonal antibody as measured by mode, D10, D50, D90 and Span (Fig. 5C). The NTA technique was thus specific, linear, accurate and precise and considered qualified to quantify particle concentration (unlabelled and labelled particles) and size distribution (unlabelled particles only as antibody interaction can change particle size although this does not impact the state of the particle as verified by TEM).

## Discussion

In this study, NTA was established as a versatile technique for characterizing RABV particle size, concentration, and antigenicity. RABV particle sizes ranged from 100 nm to 250 nm consistent with the expected size of rabies virus family^28,29^. The concentration was unaffected by anti-G D1–25 monoclonal antibody labelling, suggesting that the particles were mainly comprised of antigenic virus particles. Moreover, anti-G D1–25-labelled particle concentrations and stability measured by NTA correlated with RABV antigenicity determined by ELISA, confirming the ability of the former to characterize antigenic particles. NTA was also able to monitor the stability of antigenic RABV particles over 24 months (as with the ELISA) confirming the size, concentration and antigenicity of the RABV was constant over time, thus indicating vaccine stability. Thermal stress at 55 °C caused a decrease in the concentration of anti-G D1–25 -labelled particles from 144 hours suggesting conformational changes in the viral particles leading to a loss of G protein antigenicity without impacting the aggregation state, which directly correlated to results from the ELISA. In addition, NTA was qualified for characterization of RABV particle count and size measurements and lot-to-lot consistency was demonstrated.

The RABV particle size distribution determined in this study is consistent with that reported previously with NTA by Clénet *et al*.^21^. In addition, they showed the loss of RABV antigenicity during thermal stress assessments performed between 5 °C and 60 °C over one month by NTA mirrored the loss determined by ELISA up to 45 °C. Clénet *et al*. also observed the emergence of larger particle population sizes concomitant with decreased antigenicity during thermal stress, suggesting RABV aggregation and demonstrated that RABV particle polydispersity inversely correlated with antigenicity. Of note, in our study, at high thermal stress temperatures (55 °C) with RABV (DP) there was not a complete loss of antigenicity up to 144 hours, whereas at similar high temperatures (60 °C) there was an apparent complete loss of antigenicity within the first day, as shown previously with RABV (DS) in the Clénet study^21^. This suggests that the formulated RABV (DP) in the study has a protective element.

Although NTA may be considered the technique of choice for the characterization of polydispersed aggregate suspensions, such as with RABV, operational settings during analysis and recording may influence estimation of particle size and care should be taken to ensure that these are optimized^20,24^. It is possible that even when identical operational settings are used there may be significant divergence between instruments in estimated mean sizes and size distribution for the same biological suspension^30^. Thus, longitudinal variation in operational settings may need to be assessed using both synthetic beads and samples to validate the accuracy of the NTA equipment over time. Other potential limitations with NTA included that non-spherical particles above 500 nm may not be tracked properly by the software which could lead to smaller size estimation of the particles^24^, and that particle morphology cannot be ascertained.

In this study, as suggested by TEM, the RABV particles were relatively homogenous in terms of morphology, presenting particle sizes compatible with that expected for rabies virus family when detected by NTA. As well, results using the TEM indicate that the interaction between the antibody and the G-protein from the rabies does not alter the RABV particle and no aggregates are observed, confirming that labelled particles are in the correct conformation and suitable to be counted using the NTA method.

In conclusion, the NTA measurements in our study showed good repeatability, as demonstrated by the specificity, linearity, accuracy and precision in repeated particle count and size measurements. We also demonstrated the utility of NTA to simultaneously count and size antigenic RABV particles with the use of anti-G D1–25 monoclonal antibodies at different states of manufacturing and at different formulations. Moreover, there was good correlation between RABV antigenicity determined by NTA and that determined by the ELISA reference method, confirming the ability of NTA to characterize antigenic particles and to be a complementary technique to ELISA. We propose the use of NTA as a simple method to facilitate the characterization of RABV particle suspensions throughout the shelf life of the product.

## Material and Methods

### Cells and viruses

Samples of purified Vero Cell Rabies Vaccine (PVRV, Verorab) and PVRV-NG2 (VRVg 2.0, Sanofi Pasteur, Lyon, France) were obtained inactivated by β-propiolactone at the end of substance manufacturing process (bulk stage; drug substance [DS]). The drug product (DP) batches were obtained formulated from the inactivated bulk in three lots S4483, S4491 and S4497. RABV was formulated in stabilizer containing a mixture of excipients (amino acids, sugar, surfactant and chelator) in a phosphate buffer (pH 8) that had been reconstituted with water. DP rabies virus was incubated at 5 °C, 25 °C and 37 °C over 24 months before NTA and ELISA analysis and comparison. The thermal stability of RABV (DP) particles was also assessed in samples incubated at 55 °C for up to seven days.

### Nanoparticle tracking analysis (NTA)

All analyses were performed using a NanoSight NS300 instrument (Malvern Instruments Ltd, Worcestershire, UK), equipped with a 488 nm laser and a high sensitivity scientific complementary metal–oxide–semiconductor (sCMOS) camera from Malvern Instruments Ltd, Worcestershire, UK^21^.

Each DP sample was added to 0.5 mL of 0.4% sodium chloride, then diluted in PBS (CXXPBS00–01 Eurobio, les Ulis, France) before being analyzed by NTA. Three independent replicates for each sample (DS and DP) were analyzed and three videos of 60 seconds recorded for each. A minimum of 1,000 valid tracks were required to validate each experimental test sample. Live monitoring NTA acquisition was performed using a syringe loading system. Detection threshold (four for labelled and unlabelled particles), limiting background noise, and Camera Level (12 for unlabelled particle and 16 for labelled particle acquisitions) settings were maintained unchanged between sample acquisitions. Data were processed with NanoSight NTA 3.1 software (Malvern Instruments Ltd, Worcestershire, UK). These parameters/settings were defined in collaboration with Malvern Instruments Ltd, Worcestershire, UK.

All samples were assessed with or without anti-G D1–25 to the rabies glycoprotein conformational epitope, as previously described^25^. In brief, for the anti-G D1–25 labelled particles, RABV samples were incubated for 1 hour at 37 °C with anti-G D1–25 monoclonal antibody directly conjugated to Alexa Fluor 488 by BIOTEM (Apprieu, France), at 1:200 dilution in phosphate buffered saline. NTA data acquisition was performed using a 500 nm long pass filter to count fluorescent virus particles with bound D1–25 monoclonal antibody. Analyses of unlabelled and anti-G D1–25 labelled particles were two distinct assessments.All measurements were made according to the American Society for Testing and Materials (ASTM)^31^.

### Enzyme-linked immunosorbent assay (ELISA)

RABV antigenicity was also assessed by ELISA using two monoclonal anti-G antibodies: D1–25 and WI 1112. Monoclonal antibody W1112 was coated on ELISA plates. Plates were washed and saturation buffer added to each well and plates incubated at 37 °C for 1 h. The rabies vaccine lots were added, and plates incubated at 37 °C for 1 h. Plates were then washed and buffer containing biotin-labelled monoclonal antibody D1–25 added to each well. Plates were then washed and buffer containing streptavidin peroxidase added to each well. Plates were then washed and buffer containing revelation substrate added to each well. The OD 492 nm was determined using an ELISA reader (Molecular Devices) as previously described^25^. This ELISA was proposed to the European Directorate for the Quality of Medicines and Healthcare (EDQM) and accepted for an international collaborative study to replace the NIH activity tests for rabies vaccines^32^.

### Transmission electron microscopy (TEM)

Samples, with or without anti-G D1–25 monoclonal antibody, were negatively stained by 2% uranyl acetate as previously described^23^ and observed on a FEI Tecnai F20 electron microscope operated at 200 kV. Images were recorded with a 4k x 4k USC 4000 slow-scan CCD camera (GATAN, Inc. Pleasanton, California, United States).

### NTA qualification

The specificity, linearity, accuracy and precision of NTA for determining particle concentration and size distribution were qualified based on the Sanofi Pasteur internal procedure. Specificity was assessed using the vaccine final product matrix (i.e. not containing antigen) with or without anti-G D1–25. Linearity and accuracy was determined through triplicate NTA runs (k = 3) performed by two operators on different days. Intermediate precision was evaluated through six NTA runs (k = 6), by two operators on different days, repeated three times in order to evaluate repeatability. Particle size distributions for non-labelled particle only (as these show the particle size and the sample homogeneity), were described using five size parameters: mode (nm): size of the majority population, D10 (nm): particle diameter corresponding to 10% cumulative undersize particle size distribution, D50 (nm): the median diameter of particle size distribution, D90 (nm): the particle diameter corresponding to 90% cumulative undersize particle size distribution and Span: the distribution width which is an indicator of aggregation.

## Supplementary information

Supplementary Information.
