# Publisher Correction: Rabies Vaccine Characterization by Nanoparticle Tracking Analysis

**DOI:** 10.1038/s41598-021-82766-4

**Published:** 2021-02-01

**Authors:** M. E. Navarro Sanchez, D. Soulet, E. Bonnet, F. Guinchard, S. Marco, E. Vetter, N. Nougarede

**Affiliations:** Analytical Research & Development, Sanofi Pasteur, Campus Mérieux, 1541 Avenue Marcel Merieux, 69280 Marcy l’Etoile, France

Correction to: *Scientific Reports* 10.1038/s41598-020-64572-6, published online 18 May 2020

The original version of this Article contained a typographical error in the spelling of the author M.E. Navarro Sanchez, which was incorrectly given as Navarro Sanchez.

This has now been corrected in the PDF and HTML versions of the Article.

