# Supplementary material for: Rabies Vaccine Characterization by Nanoparticle Tracking Analysis
Source: Sci Rep. 2020 May 18;10:8149. doi: 10.1038/s41598-020-64572-6 (PMC7235079; doi:10.1038/s41598-020-64572-6)
Supplement: Supplementary file 1 — Supplementary Information. [file 41598_2020_64572_MOESM1_ESM.docx]

# Supplementary Material

**Figure S1. RABV (DP) batch consistency by NTA.** **PVRV-NG2 (VRVg 2.0)** (DP) high formulation comparison between three manufactured batches: lots S4483, S4491 and S4497 by measuring six parameters: (A) Concentration (B) Mode (C) D10 (D) D50 (E) D90 (F) Span.


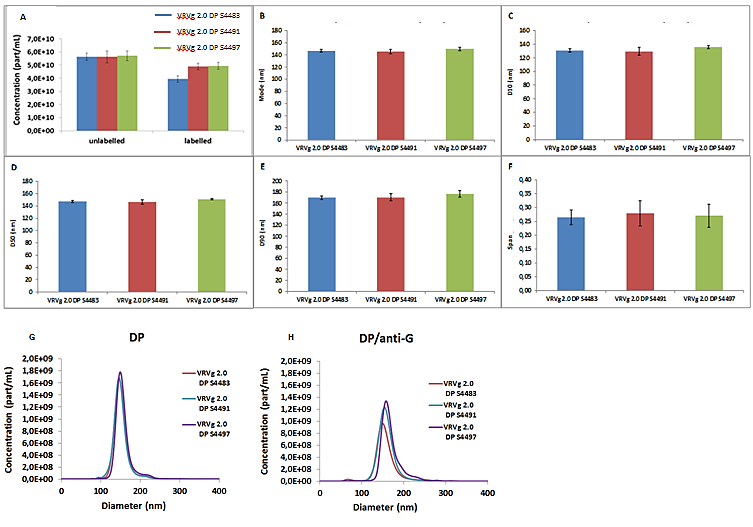


D10, nm particle diameter corresponding to 10% cumulative undersize particle size distribution; D50, nm median diameter of particle size distribution; D90, nm particle diameter corresponding to 90% cumulative undersize particle size distribution; mode, majority population size in nm; span, distribution width.DP, final product; NTA, nanoparticle tracking analysis; RABV, rabies virus

**Figure S2. Change in PVRV-NG2 (VRVg 2.0) antigenicity with thermal stress.** DP rabies virus was incubated at 55°C for up to 7 days. The antigenicity was monitored by ELISA and anti-G D1–25 monoclonal antibody label particle concentration by NTA The statistical analysis was undertaken with and without anti-G D1–25 monoclonal antibody labelling. A (RABV lot S4497) and B (RABV lot S4491) are two different high formulation DP rabies virus batches.


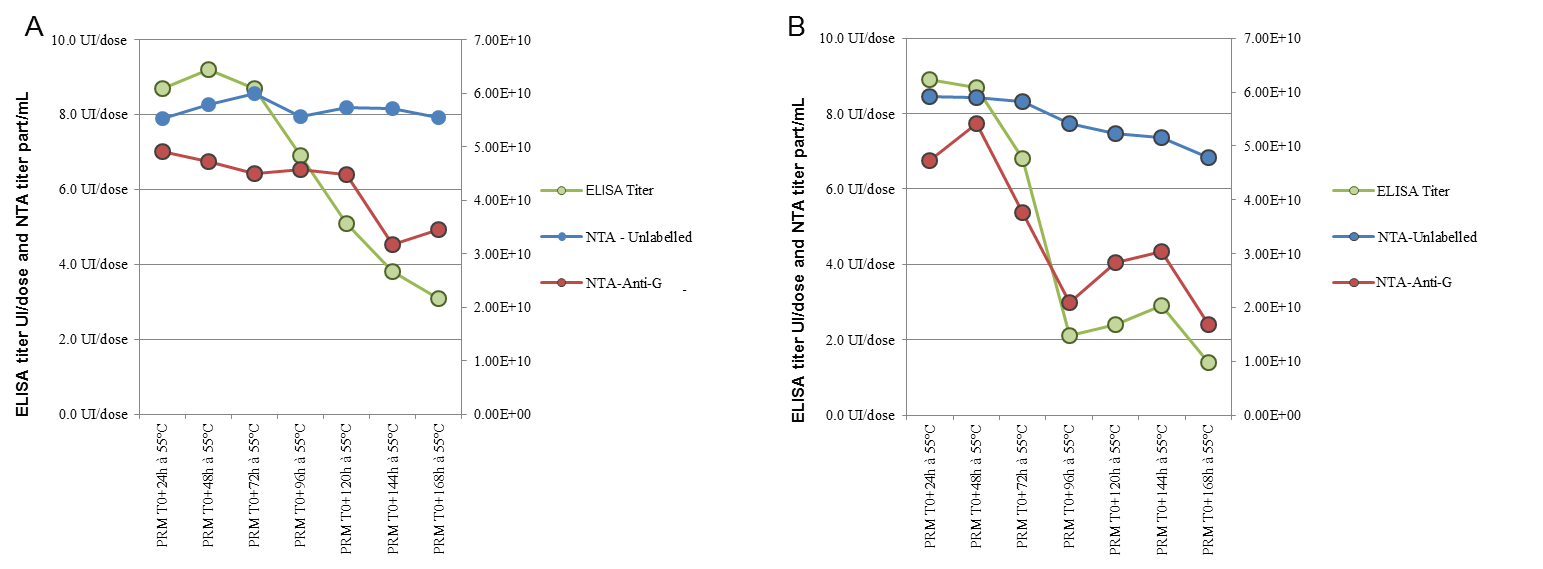


DP, final product; ELISA, enzyme-linked immunosorbent assay; NTA, nanoparticle tracking analysis; RABV, rabies virus
